# SRTdb: an omnibus for human tissue and cancer-specific RNA transcripts

**DOI:** 10.1186/s40364-022-00377-1

**Published:** 2022-04-26

**Authors:** Qili Shi, Teng Liu, Wei Hu, Zhiao Chen, Xianghuo He, Shengli Li

**Affiliations:** 1 Fudan University Shanghai Cancer Center and Institute of Biomedical Sciences, Shanghai Medical College, Fudan University, Shanghai, 200032 China; 2 Precision Research Center for Refractory Diseases, Institute for Clinical Research, Shanghai General Hospital, Shanghai Jiao Tong University School of Medicine, Shanghai, 201620 China; 3 Institute of Big Data and Artificial Intelligence in Medicine, School of Electronics and Information Engineering, Taizhou University, Taizhou, 318000 China

**Keywords:** RNA transcript, Pan-cancer analysis, Transcriptional diversity, Precision tumor diagnosis, Tissue specificity analysis

## Abstract

The production of functional mature RNA transcripts from genes undergoes various pre-transcriptional regulation and post-transcriptional modifications. Accumulating studies demonstrated that gene transcription carries out in tissue and cancer type-dependent ways. However, RNA transcript-level specificity analysis in large-scale transcriptomics data across different normal tissue and cancer types is lacking. We applied reference-based *de novo* transcript assembly and quantification of 27,741 samples across 33 cancer types, 29 tissue types, and 25 cancer cell line types. We totally identified 231,836 specific RNA transcripts (SRTs) across various tissue and cancer types, most of which are found independent of specific genes. Almost half of tumor SRTs are also tissue-specific but in different tissues. Furthermore, we found that 10 ~ 20% of tumor SRTs in most tumor types were testis-specific. The SRT database (SRTdb) was constructed based on these resources. Taking liver cancer as an example, we showed how SRTdb resource is utilized to optimize the identification of RNA transcripts for more precision diagnosis of particular cancers. Our results provide a useful resource for exploring transcript specificity across various cancer and tissue types, and boost the precision medicine for tumor patients.

**Supplementary Information:**

The online version contains supplementary material available at 10.1186/s40364-022-00377-1.

## Introduction

Over the past decade, high-throughput RNA sequencing (RNA-seq) technology has largely improved our understanding of the roles transcriptome play in various human physiological and pathological processes [1]. Transcriptome-wide analysis has become indispensable for the investigation of systematic changes in numerous aspects of RNA biology and the discovery of novel functional RNAs [2–4]. Mature RNA transcripts are the major carriers to deliver genetic codes from DNA to proteins and exert regulatory roles, which undergoes diverse pre-transcriptional regulations and post-transcriptional modifications from nascent RNA products [5]. Through alternative processing of nascent RNAs, individual genes can transcribe different RNA transcripts in distinct physiological or pathological conditions to execute specific functions [6, 7]. However, most transcriptome-wide studies have focused on gene-level activities, neglecting the specific RNA transcripts (SRTs). Emerging evidence has demonstrated the importance to determine the SRTs of genes in particular physiological and pathological conditions [8, 9].

Recently, accumulating studies have shed light on the advantage of transcript-level analysis over gene-level ones. A pilot study of alternative transcriptional isoforms across multiple human tissues revealed the universal of alternative transcriptions in different tissue types, indicating the necessity and importance of RNA transcript-level analysis [10]. In a previous study, we identified one alternative transcript of UGP2 gene, which showed significantly differential expression and indicated benign prognosis in liver cancer [9]. Zheng *et al.* analyzed approximately 1000 normal and liver cancer RNA-seq samples to identify transcripts that were exclusively expressed in liver cancer samples over normal liver samples [11]. They found that tumor-specific transcripts were frequently expressed in liver cancer and experimentally demonstrated their biological functions in liver cancer. These nonnegligible findings were masked in the stream of gene-level analysis. The specificity feature of RNA transcripts also suggests enormous potentials in tumor specific diagnosis in clinical practice.

To maximize the utility of human tissue and cancer SRTs, we conducted *de novo* transcriptome assembly of 27,741 RNA-seq samples across various tissue/cancer types and presented SRTdb (http://www.shenglilabs.com/SRTdb/), which is a comprehensive database of human tissue and cancer SRTs. In total, SRTdb database contains 1,160,216 RNA transcripts across 29 different tissue types, 33 cancer types, and 25 cancer cell line lineages. We identified 228,752, 212,214, and 231,836 SRTs in human normal tissue types, cancer types, and cell line types, respectively. Further analysis revealed that tissue/cancer SRTs are independent of corresponding specific genes (SRGs) and about half of tumor SRTs are also specific in other normal tissues, especially the testis. Overall, our results offered a panorama of RNA transcript specificity across various tissue and cancer types, and laid a solid data foundation for cancer precision medicine.

## Materials and methods

### RNA-seq data collection

The RNA-seq read alignments (BAM files) of 16,367 human normal tissue samples from 29 different tissue types were downloaded from the Genotype-Tissue Expression data portal (GTEx, https://www.gtexportal.org/) with official authorization. The RNA-seq read alignments (BAM files) of 10,358 human tumor samples from 33 different cancer types were obtained from the Genomic Data Commons data portal (GDC, https://portal.gdc.cancer.gov/) with official authorization. The raw RNA-seq data (FASTQ files) of 1016 human cancer cell lines from 25 different primary sites were downloaded from the Sequence Read Archive (SRA, https://www.ncbi.nlm.nih.gov/sra) database with accession of SRP186687. These data were released by the Cancer Cell Line Encyclopedia project (CCLE, https://portals.broadinstitute.org/ccle/). Raw sequencing reads were aligned to the human reference genome (GRCh38) by using STAR software to generate read alignments for each cell line.

### Transcript assembly and quantification

Individual read alignment files were provided as input to StringTie [12] for *de novo* transcript assembly. Transcript annotation from GENCODE version 22 was used as the transcript model reference to guide the assembly process with “-G” option. Transcript assembly was performed separately in each sample. Then all assembled transcripts were merged to generate a nonredundant master set of transcripts for all samples by using the “--merge” mode of StringTie. StringTie quantification was utilized to produce transcript-level expression for each sample. Expression levels were normalized in TPM units (TPM = Transcripts Per Million mapped reads). In each tissue/cancer/cell type, transcripts with expression levels higher than 0.1 TPM in at least one sample were remained as expressed transcripts.

### Calculation of expression specificity scores

To obtain tissue/cancer/cell type-specific transcripts, a specificity score was calculated for each transcript, which was described in our previous study [9]. In particular, the specificity score was equal to the logarithm of lineage number minus Shannon entropy of transcript expression. The calculation is as follows:

$${S}_t={\log}_2(N)-\left(-{\sum}_{i=1}^N\left({p}_{it}\times {\log}_2{p}_{it}\right)\right)$$

where *S*_*t*_ represents specificity score of transcript *t*, *N* is the total number of tissue/cancer/cell types, *p*_*it*_ indicates the expression ratio of transcript *t* in tissue/cancer/cell type *i*. One specificity score and *N* expression ratio were assigned to each transcript. The expression ratio of each transcript across all tissue/cancer/cell types is calculated as follows:

$${p}_{it}=\frac{x_{it}}{\sum_{i=1}^N{x}_{it}}$$

where *p*_*it*_ is the expression ratio of transcript *t* in tissue/cancer/cell type *i*, *N* indicates the total number of tissue/cancer/cell types, *x*_*it*_ represents the expression value of transcript *t* in tissue/cancer/cell type *i*. When the largest expression ratio is more than two times compared to the second largest expression ratio and specificity score is larger than 1, the transcript was defined as tissue/cancer/cell type-specific transcript in the tissue/cancer/cell type with largest expression ratio.

### Calculation of specific diagnostic scores in different tumor types

To further filter out transcripts specific for individual tumor types, we calculated one specific diagnostic score for each tumor SRT by integrating the expression level, tumor specific scores, and tissue specific scores. Only transcripts that were expressed in tumor samples but not the corresponding normal samples were used to calculated specific diagnostic scores. Specific diagnostic scores are calculated as follows:

$${S}_{tc}={\beta}_1\times {\log}_2\left({x}_{tc}+1\right)\times {s}_{tc}\times {r}_{tc}+{\beta}_1\times {\beta}_2\times {s}_{tt}$$

where *S*_*tc*_ is the specific diagnostic score of transcript *t* in cancer type *c*, *x*_*tc*_ indicates the average expression level of transcript *t* across tumor samples in cancer type *c*, s_*tc*_ is the specificity score of transcript *t* in cancer type *c*, *r*_*tc*_ represents the expression frequency of transcript *t* across samples in cancer type *c*, *s*_*tt*_ is the tissue specific score of transcript *t* in tissue *t*, *s*_*tt*_ is set to 1 when transcript *t* is not a tissue-specific transcript. β_1_ and β_2_ are weight coefficients. β_1_ is set to 1 when transcript *t* is a specific transcript in cancer type *c*, β_1_ is set to −1 when transcript *t* is specific in other cancer types. β_2_ is set to 1 when transcript t isa specific transcript in tissue type *t*, β_2_ is set to 0 when transcript *t* is specific in other tissue types. The larger *S*_*tc*_ value is, the higher reliability of transcript *t* as a specific diagnostic biomarker in cancer type *c* is.

### Protein-coding potential prediction of unannotated transcripts

CPAT (http://lilab.research.bcm.edu/cpat/) [13] and CPC2 [14] (http://cpc2.cbi.pku.edu.cn/) were employed to predict the protein-coding potential of unannotated transcripts. Coding potential scores of transcripts in CPAT that were larger than the default value of 0.364 were labeled as protein-coding. The transcript was considered with coding potential when it was predicted in both two algorithms.

### Database and web site implementation

SRTdb database was built with Python FLASK_REST API (https://flask-restful.readthedocs.io/) as backend web framework. In SRTdb database, MongoDB (https://www.mongodb.com) was adopted for data deposition and management. Angular (https://angular.io/) was utilized to develop web interfaces of SRTdb. The frontend framework was constructed by using Bootstrap (https://getbootstrap.com). Data visualization was carried out by Echarts (https://echarts.apache.org/). The SRTdb online database is tested and supported in popular web browsers, including Microsoft Edge, Google Chrome, Firefox, and Safari.

## Results

### SRTdb is resourced from over 27,000 human RNA-seq samples

**Fig. 1:**
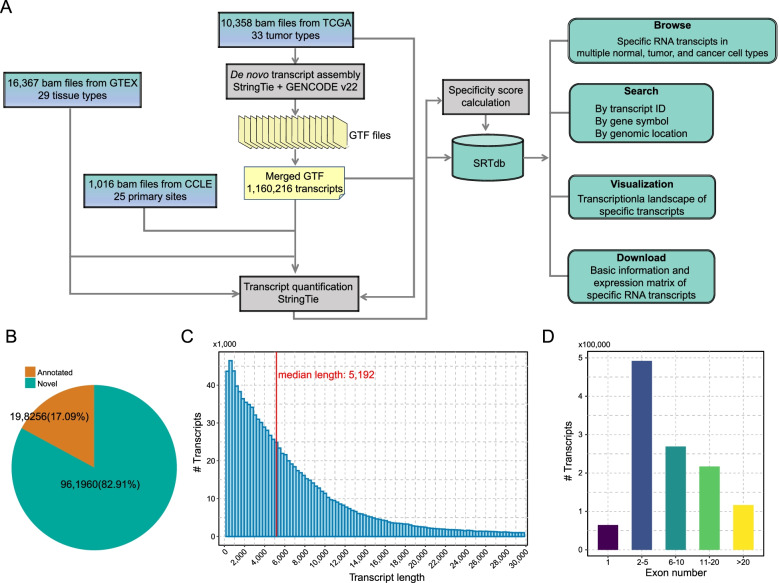
The infrastructure of online SRTdb database. **A** Transcripts were generated from *de novo* transcript assembly of 10,358 tumor samples across 33 different tumor types and 1,160,216 transcripts were totally identified. Transcript quantification was also performed in 16,367 normal tissue samples across 29 different tissue types and 1016 cancer cell lines across 25 different primary sites. Tumor, tissue, and cancer cell type-specific scores were calculated. SRTdb offers features of browse, search, visualization, and download for all users. **B** Piechart shows the percentages of annotated and novel transcripts. **C** The distribution of transcript length. **D** The distribution of exon numbers in transcripts

SRTdb is a database aiming to collect and annotate human specific transcript RNAs, especially cancer-specific transcripts, from large-scale RNA-seq datasets. Currently, SRTdb analyzed 27,741 samples across 33 tumor types, 29 normal tissue types, and 25 cancer cell lineages (Table 1). To make the transcript identification more sensitive to tumor, we first performed refence-based *de novo* transcriptome assembly across 10,358 tumor samples (Fig. 1A). Briefly, *de novo* transcript assembly was separately conducted in individual tumor samples (see Materials and Methods). Then, all assembled transcripts were merged to generate one non-redundant set of transcripts as the master transcript annotation for following analyses. In total, 1,160,216 transcripts were identified, of which 198,256 are annotated transcripts and 961,960 (82.91%) are novel transcripts (Fig. 1B). The length of identified transcripts ranged widely from 200 bp to 30 kb, with median length of 5192 bp (Fig. 1C). About 40% of transcripts are composed by 2 ~ 5 exons, and approximately 10% have more than 20 exons (Fig. 1D). Among the novel transcripts, 96.1% (924,470/961,960) are multi-exon transcripts, indicating that these transcripts might undergo post-transcriptional processing, such as alternative splicing. In addition, most of the novel transcripts were non-coding, and only 2% of the transcripts were predicted to have protein-coding potential. These transcripts were then quantified in 16,367 normal tissue samples and 1016 cancer cell line samples. By median numbers, 248,832, 294,601, and 299,394 transcripts were identified expressed in tumor, normal tissues, and cancer cell lines, respectively (Supplemental Table S1). In tumor, the largest number of transcripts were detected in stomach cancer (i.e., 358,830), while uveal melanoma samples express the smallest number of transcripts (i.e., 183,267). The testis tissue shows the highest levels of transcriptional diversity with 427,963 different transcripts expressed, whereas muscle tissue expresses the lowest amount with 209,973 transcripts. Cell lines derived from biliary tract tumor expressed the most transcripts (i.e., 351,217), while only 147,731 transcripts were detected transcriptionally active in small intestine cancer-derived cell lines. Based on expression profiles of transcripts from over 27,000 samples, expression specificity scores were calculated to identify specific RNA transcripts across different tumor types, normal tissue types, and cancer cell line types.

**Table 1:** The numbers of samples in each cancer, tissue, and cell line type

Type	Normal tissue	Tumor tissue^a^	Cancer cell line
Adipose tissue	1204	0	0
Adrenal gland	258	79 (ACC)	0
Bladder	21	415 (BLCA)	0
Blood	929	151 (LAML)	0
Blood vessel	1335	0	0
Brain	1668	530 (LGG);169 (GBM)	65
Breast	457	1109 (BRCA)	57
Cervix	19	302 (CESC)	3
Colon	779	480 (COAD)	59
Esophagus	1434	160 (ESCA)	26
Heart	857	0	0
Kidney	89	289 (KIRP); 539 (KIRC); 65 (KICH)	32
Liver	226	372 (LIHC)	25
Lung	573	536 (LUAD); 502 (LUSC)	191
Muscle	799	0	0
Nerve	619	183 (PCPG)	16
Ovary	180	379 (OV)	47
Pancreas	328	178 (PAAD)	41
Pituitary	283	0	0
Prostate	242	500 (PRAD)	8
Salivary gland	162	0	2
Skin	1806	471 (SKCM)	56
Small intestine	187	0	1
Spleen	241	0	0
Stomach	359	375 (STAD)	37
Testis	361	156 (TGCT)	0
Thyroid	653	509 (THCA)	11
Uterus	142	56 (UCS); 552 (UCEC)	27
Vagina	156	0	0
Biliary tract	0	36 (CHOL)	8
Soft tissue	0	263 (SARC)	31
Lymphoma	0	48 (DLBC)	176
Head and Neck	0	502 (HNSC)	32
Mesothelium	0	86 (MESO)	0
Rectum	0	167 (READ)	0
Thymus	0	119 (THYM)	0
Eye	0	80 (UVM)	0
Bone	0	0	28
Pleura	0	0	11
Urinary tract	0	0	26

^a^*ACC* adrenocortical carcinoma, *BLCA* Bladder Urothelial Carcinoma, *LAML* Acute Myeloid Leukemia, *LGG* Brain Lower Grade Glioma, *GBM* Glioblastoma multiforme, *BRCA* Breast invasive carcinoma, *CESC* Cervical squamous cell carcinoma and endocervical adenocarcinoma, *COAD* Colon adenocarcinoma, *ESCA* Esophageal carcinoma, *KIRP* Kidney renal papillary cell carcinoma, *KIRC* Kidney renal clear cell carcinoma, *KICH* Kidney Chromophobe, *LIHC* Liver hepatocellular carcinoma, *LUAD* Lung adenocarcinoma, *LUSC* Lung squamous cell carcinoma, *PCPG* Pheochromocytoma and Paraganglioma, *OV* Ovarian serous cystadenocarcinoma, *PAAD* Pancreatic adenocarcinoma, *PRAD* Prostate adenocarcinoma, *SKCM* Skin Cutaneous Melanoma, *STAD* Stomach adenocarcinoma, *TGCT* Testicular Germ Cell Tumors, *THCA* Thyroid carcinoma, *UCS* Uterine Carcinosarcoma, *UCEC* Uterine Corpus Endometrial Carcinoma, *CHOL* Cholangiocarcinoma, *SARC* Sarcoma, *DLBC* Lymphoid Neoplasm Diffuse Large B-cell Lymphoma, *HNSC* Head and Neck squamous cell carcinoma, *MESO* Mesothelioma, *READ* Rectum adenocarcinoma, *THYM* Thymoma, *UVM* Uveal Melanoma

### Exploring tumor and tissue-specific RNA transcripts with SRTdb

**Fig. 2:**
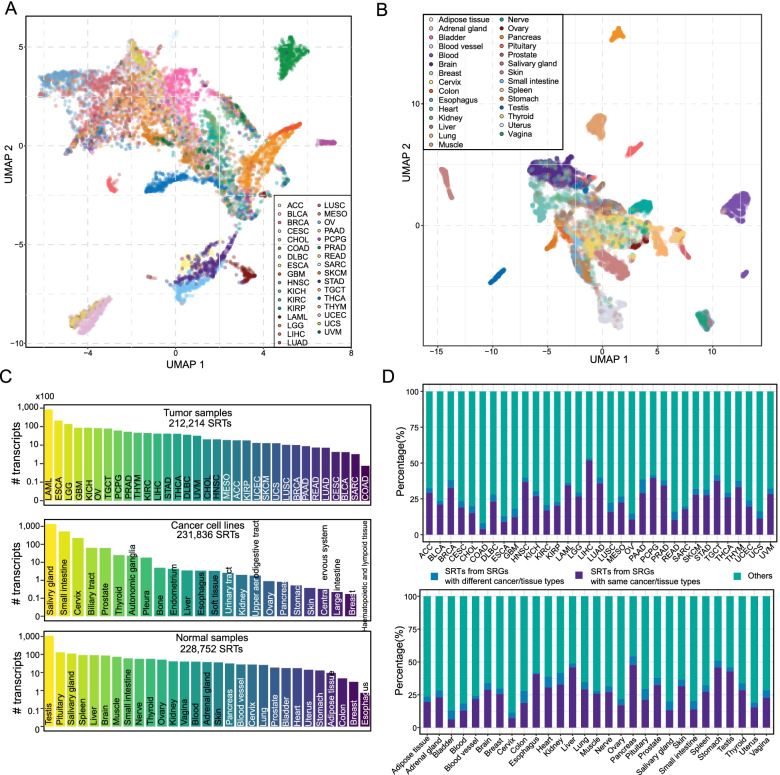
Exploration of specific RNA transcripts with the SRTdb resource. **A** UMAP visualization of tumor samples by using top 2000 variable transcripts. **B** UMAP visualization of normal samples by using top 2000 variable transcripts. **C** The number distributions of SRTs across different tumor types, cancer cell line types, and normal tissue types. **D** The percentages of SRTs in host SRGs across tumor (top panel) and normal tissue (bottom panel) samples

The expression profiles of transcripts were adopted to cluster samples separately in tumor and normal samples by using top 2000 variable transcripts. Transcript expression profiles showed notable performance to distinguish different tumor types (Fig. 2A) and normal tissue types (Fig. 2B). To identify exclusively expressed transcripts in individual tumor types, normal tissue types, and cancer cell line types, expression specificity scores were calculated by employing a method of Shannon entropy (see Materials and Methods). In the current version of SRTdb, we totally identified and curated 212,214, 231,836, and 228,752 SRTs in tumors, cancer cell lines, and normal tissues, respectively. The number of transcripts specific to each cancer type varies considerably (Fig. 2C). The most specifically expressed transcripts are found in acute myeloid leukemia (LAML), followed by esophageal carcinoma (ESCA), brain lower grade glioma (LGG) and glioblastoma multiforme (GBM). Notably, there are fewer SRTs for cancers of the same tissue type, such as lung adenocarcinoma (LUAD) and lung squamous carcinoma (LUSC), both of which are derived from lung tissue. Cancers of colorectum, colon adenocarcinoma (COAD) and rectum adenocarcinoma (READ), also have fewer SRTs. The major reason for these results is that cancers of the same or related tissue types have relatively similar expression patterns. A certain number of specifically expressed transcripts are present in each normal tissue type, and these transcripts may be tightly related to tissue-specific functions. The testis has the highest number of specifically expressed RNAs, followed by pituitary, salivary gland, spleen and liver. These SRTs can be employed as molecular biomarkers for different tissue types. Similarly, distinct numbers of specific transcripts were obtained in different cancer cell line types. To examine whether specific transcripts are independent of specific genes, we also evaluated the specificity of corresponding host genes. In most cancer and tissue types, the majority of SRTs are not from specific genes (Fig. 2D). This result demonstrated that a large portion of valuable RNA transcripts were neglected by the gene-level analysis.

The SRTdb data portal mainly includes “Browse”, “Search”, and “Download” features. Through a user-friendly interface, users can browse the SRTs by selecting a specific tumor, cancer cell line, or normal tissue type from the dataset column on the left (Supplementary Fig. S1A), and the result will be displayed in a table on the right. The result table contains transcript ID, specificity score, specificity ratio, specific cancer type, tissue type, gene symbol, genomic loci, sequence, length, and transcript type. By clicking the transcript ID, users will be redirected to a new web page showing the transcript basic information, expression specificity and boxplots of expression profiles across cancer types, normal tissues, and cancer cell lines (Supplementary Fig. S1B). Users can query transcripts of interest by transcript ID, gene name, or genomic loci on the “Search” page (Supplementary Fig. S1C). In addition, users can also do quick search by gene symbol in the homepage. Files of transcript annotation and specificity can be downloaded from the “Download” page.

### Specificity analysis reveals dual roles of tumor SRTs

**Fig. 3:**
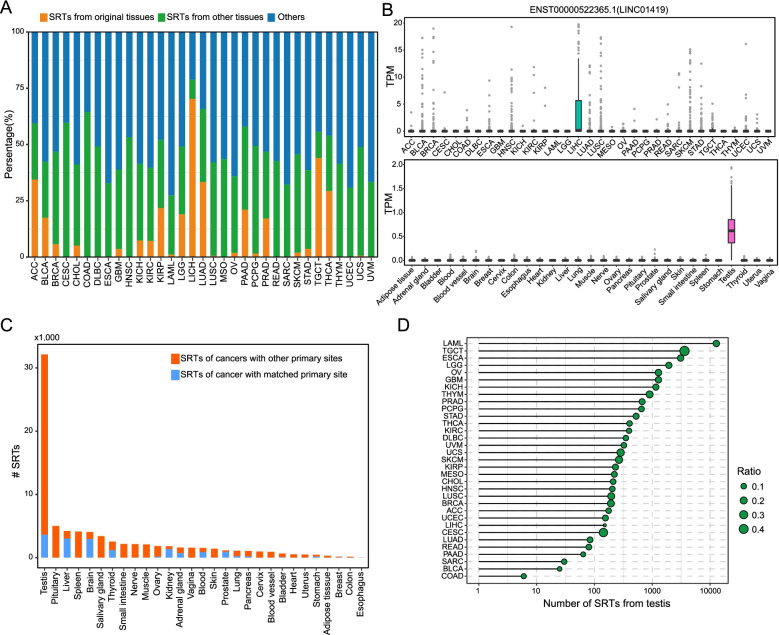
The tissue origins of tumor SRTs. **A** The percentages of tumor SRTs specific in original tissue types, other tissue types or not tissue-specific. **B** The expression distribution of liver cancer-specific transcript, ENST00000522365.1, across multiple cancer and normal tissue types. **C** The number distribution of tissue SRTs specific in matched tumor types, other cancer types or not cancer-specific. **D** The numbers of testis SRTs across different tumor types

To further explore the potential clinical utility of tumor SRTs, we examined their specificity distribution across multiple tumor and tissue types. In average, approximately half of tumor SRTs are also tissue SRTs (Fig. 3A). Interestingly, the majority of tumor SRTs are exclusively expressed in tissue types that are not the particular tumor types where SRT originate. For example, one of LINC01419 transcripts, ENST00000522365.1, is specifically expressed in liver cancer across multiple cancer types, while exclusively expresses in testis tissue across various tissue types (Fig. 3B). In some tumor types, the vast majority of tumor SRTs are also specific in original tissues, especially liver cancer (Supplemental Fig. S2A). The transcript of APOA2, ENST00000367990.6, is exclusively active in liver cancer across different cancer types, and also specifically expressed in liver tissue across multiple tissue types (Supplemental Fig. S2B). To further explore the specificity distribution of transcripts, we examined the tumor specificity of tissue SRTs. Strikingly, more than 30,000 testis SRTs are also specific in tumors, wherein most are specific in tumor types originating from tissues other than the testis (Fig. 3C). We next examined how many tumor SRTs are testis-specific in each tumor type. In all tumor types, tumor SRTs from LAML constitute the largest population of testis SRTs, followed by TGCT, ESCA, and LGG (Fig. 3D). In most tumor types, 10 ~ 20% of tumor SRTs are testis-specific. As expected, the most portion (about 40%) of TGCT tumor SRTs are also testis SRTs. CESC has the second largest percent (about 30%) of tumor SRTs that are testis-specific, which may be due to reproductive functions.

### The application of SRTdb for precision cancer diagnosis: liver cancer as an example

**Fig. 4:**
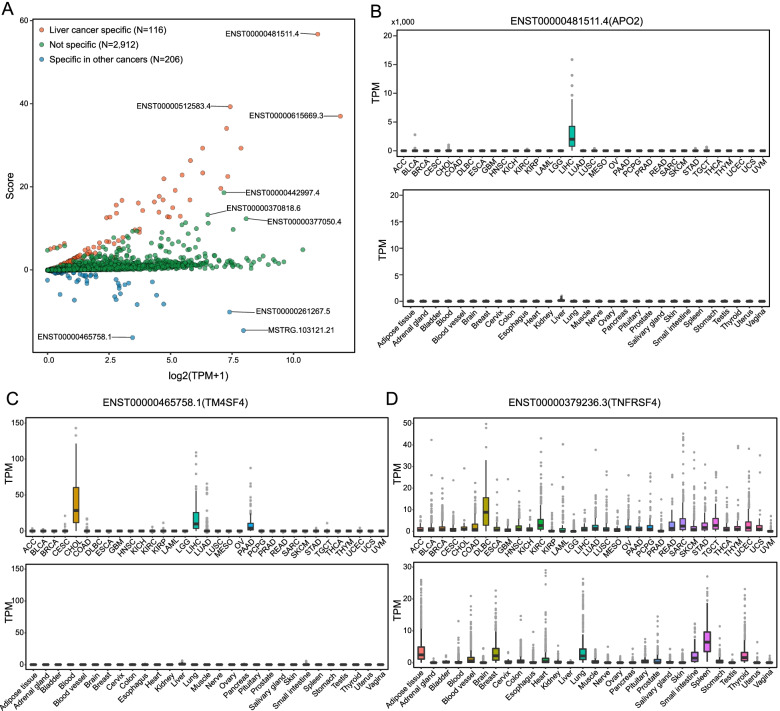
The diagnostic value of liver cancer-specific transcripts. **A** The specific diagnostic scores of transcripts for liver cancer. The expression distribution of transcript ENST00000481511.4 (**B**), ENST00000465758.1 (**C**), and ENST00000379236.3 (**D**) across different tumor and normal tissue types

A considerable part of tumor SRTs were found to be specifically expressed in particular normal tissues, disclosing possibly promiscuous assignment of specific RNAs in pan-cancer or paired tumor-normal studies. The SRT resources deposited in SRTdb data portal can be used to develop specific diagnostic RNA markers in particular cancer types. Here, we took liver cancer as an example to show the application of SRTdb database for precision diagnosis of cancer. Liver cancer transcripts were first filtered to discard transcripts that were expressed at considerable level in normal tissues. Then a specific diagnostic score was calculated for each of filtered liver cancer SRTs (see Materials and Methods). Compared to normal tissues, 3234 transcripts were specifically expressed in liver cancer, wherein only 116 were stringently liver cancer-specific (Fig. 4A). For example, one of APO2 transcripts, ENST00000481511.4, is exclusively expressed in liver cancer across different cancer types, and showed negligible transcriptional activity across multiple normal tissues, including normal liver tissue (Fig. 4B). The detection of ENST00000481511.4 in any sample from liquid biopsy or liver tissue very likely indicate the occurrence of liver cancer. Although ENST00000465758.1 (one of TM4SF4 transcripts) is specifically expressed in liver cancer over normal liver tissue and any other tissue, it is also detected transcriptionally active in other cancer types, such as cholangiocarcinoma and pancreas cancer (Fig. 4C). In another case, the transcript ENST00000379236.3 (from the TNFRSF4 gene) expressed much higher in liver cancer over normal liver tissue, but also showed considerable expression level in other cancer and tissue types (Fig. 4D). These results demonstrated the necessity of examining transcript-level expression across different tumor and tissue types for precision tumor diagnosis.

## Discussion

In the recent decade, RNA-seq techniques have unearthed the vast diversity of transcriptome and their applications in cell/tissue identity and clinical diagnosis/treatment [1, 15, 16]. Nevertheless, studies have focused on gene levels. For a long time, one genomic location of a particular gene has been deemed to transcribed one single or major RNA transcript [17]. Although experimental validations are based on RNA transcripts, a large portion of isoform transcripts have been ignored. With the development of RNA-seq techniques and computational algorithms, more and more transcriptional isoforms and their functions have been discovered [18, 19]. To unveil the transcriptional diversity and specificity across multiple human tissues and tumors, we conducted reference-based *de novo* transcript assembly and quantification of 27,741 samples across 29 tissue types, 33 cancer types, and 25 cancer cell line types. Our results revealed dual roles of tumor SRTs, wherein tumor SRTs also showed exclusive expression in particular tissues. We presented a publicly accessible data portal, SRTdb, to facilitate the exploration of cancer transcriptome and transcriptional specificity at transcript resolution. Efforts have been made to identify specific transcriptional activities across various tissue or cancer types [20–22]. But these results were based on gene-level quantification, which failed to consider the ubiquitous transcriptional isoforms. To minimize the effect of batch effects between samples, we applied a widely used RNA-seq pipeline to process all the samples [9, 23, 24]. All the RNA-seq samples went through the same alignments, transcript quantification and expression normalization.

Some published databases also provided RNA transcript information in human tissues or cancers, such as cncRNAdb [25], NONCODE [26], and NoncoRNA [27], but they are quite different from the SRTdb database. The cncRNAdb database collected about 2000 experimentally supported cncRNAs (coding and noncoding RNAs) across over 20 species. The NoncoRNA database curated 5568 experimentally supported non-coding RNAs and their drug target associations in cancer. Both cncRNAs and NoncoRNA only provided gene-level but no specific transcript information of RNAs, and they didn’t include expression levels across different human tissues and cancers. The NONCODE database provided integrated knowledge of noncoding RNAs across 39 different species. Although NONCODE provided the information of specific transcripts of RNAs, it is different from our SRTdb database in three major aspects: it focuses on noncoding RNAs; it doesn’t provide RNA specificity across human tissues and cancers; these RNA transcripts were retrieved from published papers or databases.

Precision diagnosis of cancer is vital for preventing further deterioration and developing treatment strategies. Specificity of diagnostic markers or factors is crucial for differential or precision diagnosis, which includes discrimination from both normal tissues and other cancer types. Diagnostic specificity is especially important in non-invasive diagnosis, such as liquid biopsy, which is one of the most important means in the early detection of cancers [28, 29]. Our results found that most tumor SRTs were also specifically expressed in other tumor types or particular normal tissues, indicating possible misdiagnosis in liquid biopsy. By utilizing the SRTdb resource, we also developed a specific diagnosis score system to identify transcripts of precision diagnosis of particular cancer types.

The advent of the third generation of RNA sequencing (TGRS) technologies (i.e., long-read or full-length RNA sequencing) has expedited the more accurate identification of full-length RNA transcripts [30]. TGRS will rectify a variety of RNA transcripts that were assembled from RNA-seq data by computational algorithms. Even though, major findings from computationally assembled RNA transcripts will largely promote the development of precision cancer medicine. The SRTdb data portal presented in this study is expected to assist our deeper understanding of cancer transcriptome diversity and precision cancer diagnosis in the clinical practice.

## Supplementary Information

**Additional file 1: Supplemental Table S1.** The numbers of expressed transcripts in each tumor, normal tissue, and cancer cell line type. **Supplemental Figure S1.** Brief introduction of how to use SRTdb database. **Supplemental Figure S2.** The expression distribution of liver cancer SRTs.

## Data Availability

All results and data described in this study, including basic information of all transcripts, specificity scores of transcripts, and expression profiles of transcripts in tumors, normal tissues, and cancer cell lines, are available at http://www.shenglilabs.com/SRTdb.
