# Supplementary material for: SRTdb: an omnibus for human tissue and cancer-specific RNA transcripts
Source: Biomark Res. 2022 Apr 26;10:27. doi: 10.1186/s40364-022-00377-1 (PMC9044872; doi:10.1186/s40364-022-00377-1)
Supplement: Supplementary file 1 — Additional file 1: Supplemental Table S1. The numbers of expressed transcripts in each tumor, normal tissue, and cancer cell line type. Supplemental Figure S1. Brief introduction of how to use SRTdb database. Supplemental Figure S2. The expression distribution of liver cancer SRTs. [file 40364_2022_377_MOESM1_ESM.docx]

**Supplemental Materials**

**Supplemental Tables**

**Supplemental Table S1. The numbers of expressed transcripts in each tumor, normal tissue, and cancer cell line type.**

| **Type** | **Normal tissue** | **Tumor** | **Cancer cell line** | | |
| --- | --- | --- | --- | --- | --- |
| Adipose tissue | 293,673 | NA | | NA |  |
| Adrenal gland | 267,058 | 210,500 (ACC); | | NA |  |
| Bladder | 287,709 | 245,863 (BLCA) | | NA |  |
| Blood | 210,350 | 329,234 (LAML) | | NA |  |
| Blood vessel | 283,084 | NA | | NA |  |
| Brain | 282,783 | 296,140 (GBM);  278,735 (LGG) | | NA |  |
| Breast | 311,243 | 264,150 (BRCA) | | 301,861 |  |
| Cervix | 345,538 | 250,164 (CESC) | | 225,418 |  |
| Colon | 295,529 | 237,623 (COAD) | | 307,629 |  |
| Esophagus | 287,016 | 265,715 (ESCA) | | 290,302 |  |
| Heart | 227,884 | NA | | NA |  |
| Kidney | 271,558 | 248,832 (KIRP); 277,546 (KIRC);  217,265 (KICH) | | 284,082 |  |
| Liver | 219,259 | 206,864 (LIHC) | | 299,863 |  |
| Lung | 322,621 | 265,629 (LUAD);  273,431 (LUSC) | | 296,032 |  |
| Muscle | 209,973 | NA | | NA |  |
| Nerve | 322,807 | 235,126 (PCPG) | | 316,820 |  |
| Ovary | 299,749 | 311,816 (OV) | | 304,484 |  |
| Pancreas | 211,923 | 258,018 (PAAD) | | 290,060 |  |
| Pituitary | 326,387 | NA | | NA |  |
| Prostate | 315,272 | 243,817 (PRAD) | | 339,516 |  |
| Salivary gland | 303,553 | NA | | 235,013 |  |
| Skin | 303,433 | 241,500 (SKCM) | | 302,675 |  |
| Small intestine | 318,410 | NA | | 147,731 |  |
| Spleen | 292,509 | NA | | NA |  |
| Stomach | 269,842 | 358,830 (STAD) | | 306,164 |  |
| Testis | 427,963 | 273,591 (TGCT) | | NA |  |
| Thyroid | 327,549 | 241,183 (THCA) | | 271,183 |  |
| Uterus | 307,153 | 256,323 (UCEC);  254,325 (UCS) | | NA |  |
| Vagina | 313,473 | NA | | NA |  |
| Biliary tract | NA | 258,586 (CHOL) | | 351,217 |  |
| Soft tissue | NA | 247,948 (SARC) | | 281,088 |  |
| Lymphoma | NA | 231,233 (DLBC) | | 300,726 |  |
| Head and Neck | NA | 234,945 (HNSC) | | 278,251 |  |
| Mesothelium | NA | 242,313 (MESO) | | NA |  |
| Rectum | NA | 238,920 (READ) | | NA |  |
| Thymus | NA | 241,183 (THYM) | | NA |  |
| Eye | NA | 183,267 (UVM) | | NA |  |
| Bone | NA | NA | | 320,005 |  |
| Pleura | NA | NA | | 259,249 |  |
| Urinary tract | NA | NA | | 299,394 |  |

**Supplemental Figures**


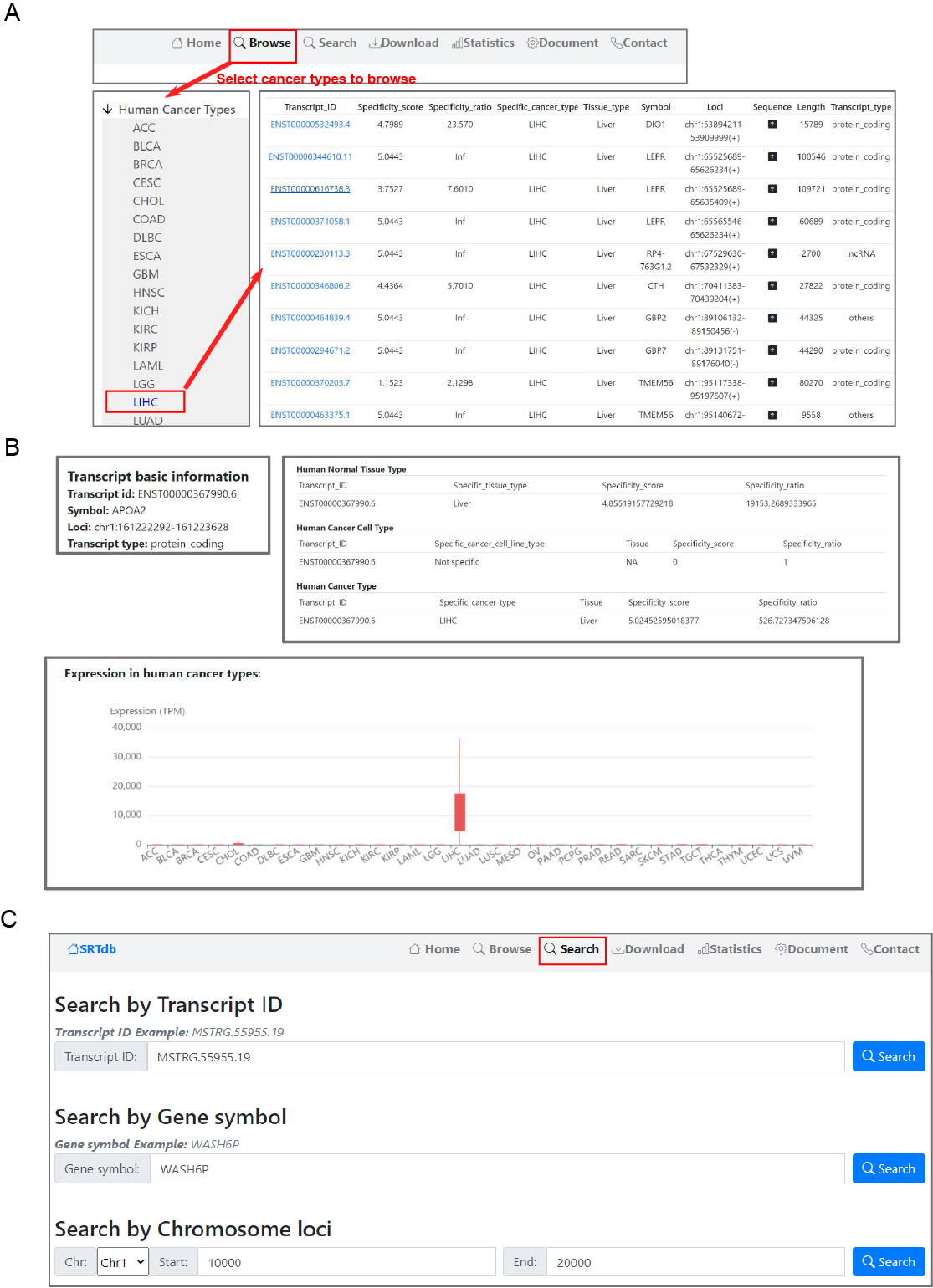


**Supplemental Figure S1. Brief introduction of how to use SRTdb database.** (**A**) Browse the transcript data by tumor/tissue/cancer cell line types in SRTdb data portal. (**B**) The basic information and expression distribution across multiple tumor types of the queried transcript. (**C**) Search transcripts of interest by transcript ID, gene symbol, and chromosome loci in SRTdb.


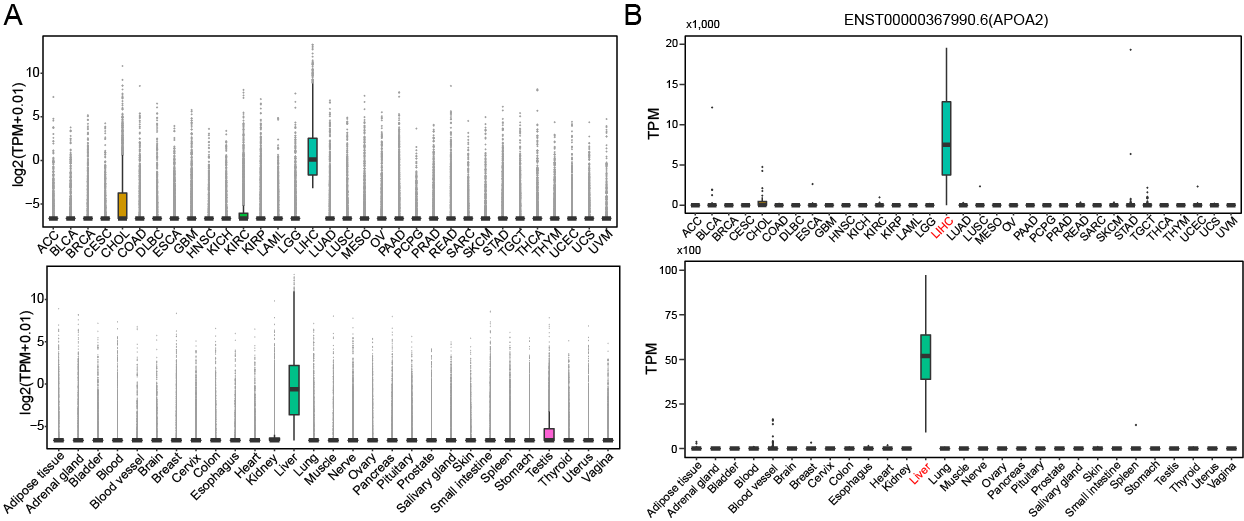


**Supplemental Figure S2. The expression distribution of liver cancer SRTs.** (**A**) The overall expression distribution of liver cancer SRTs across different cancer and tissue types. (**B**) The expression distribution of transcript ENST00000367990.6 across various tumor and tissue types.
